# Index Surgery Cost of Fluoroscopic Freehand Versus Robotic-Assisted Pedicle Screw Placement in Lumbar Instrumentation: An Age, Sex, and Approach-Matched Cohort Comparison

**DOI:** 10.5435/JAAOSGlobal-D-22-00137

**Published:** 2022-12-02

**Authors:** Ekene Uchenna Ezeokoli, Mitchell Pfennig, Jithin John, Rohun Gupta, Jad G. Khalil, Daniel K. Park

**Affiliations:** From the Oakland University William Beaumont School of Medicine, Rochester, MI (Ezeokoli, Pfennig, John, and Gupta), and the Department of Orthopaedic Surgery, Beaumont Health Systems, Royal Oak, MI (Dr. Khalil and Dr. Park).

## Abstract

Spine surgery costs are notoriously high, and there are already criticisms and concerns over the economic effects. There is no consensus on cost variation with robot-assisted spine fusion (rLF) compared with a manual fluoroscopic freehand (fLF) approach. This study looks to compare the early costs between the robotic method and the freehand method in lumbar spine fusion.

**Methods:**

rLFs by one spine surgeon were age, sex, and approach-matched to fLF procedures by another spine surgeon. Variable direct costs, readmissions, and revision surgeries within 90 days were reviewed and compared.

**Results:**

Thirty-nine rLFs were matched to 39 fLF procedures. No significant differences were observed in clinical outcomes. rLF had higher total encounter costs (*P* < 0.001) and day-of-surgery costs (*P* = 0.005). Increased costs were mostly because of increased supply cost (0.0183) and operating room time cost (*P* < 0.001). Linear regression showed a positive relationship with operating room time and cost in rLF (*P* < 0.001).

**Conclusion:**

rLF is associated with a higher index surgery cost. The main factor driving increased cost is supply costs, with other variables too small in difference to make a notable financial effect. rLF will become more common, and other institutions may need to take a closer financial look at this more novel instrumentation before adoption.

Robotic-assisted procedures are becoming increasingly common in orthopaedics, especially in total knee arthroplasty and spinal instrumentation. Pedicle screws are an established and widely accepted method used for spinal fixation for the treatment of deformities, traumas, and neoplasms of the thoracolumbar spine.^1^ Robot-guided pedicle screw placement is meant to provide improved accuracy and precision in pedicle screw placement along with a reduction in exposure to radiation for the surgeon, patient, and operating room (OR) staff.^2,3,4,5,6,7,8,9,10,11,12,13,14^ Several robotic systems have found recent increased usage in spinal instrumentation, most notably MAZOR X (Medtronic Navigation).^15^ Evidence that the use of robotics will lead to improved survival, function, and patient-reported outcomes and decreased complications is not consistent, and while there is some evidence of benefits over a fluoroscopic approach, many studies have shown equivalent or variable outcomes.^16,17,18,19^

Spine surgery costs are notoriously high, and there are already criticisms and concerns over the economic effects.^20,21^ Some studies have looked at overall costs, costs of different approaches to the spine, and geographic variation in costs;^22-24^ however, there are few studies directly evaluating costs in robotic-assisted spine surgeries compared with more manual nonrobotic techniques. Passias et al^25^ looked at cost in robotic-assisted cases compared with matched minimally invasive surgery (MIS) and open cases and found markedly higher costs with the robot. By contrast, Menger et al^26^ found robotic-assisted cases to be cost-effective, and Garcia et al^27^ suggested cost-effectiveness to be dependent on individual OR and institution admission costs.

To date, there is still minimal and conflicting literature investigating the cost of robotic-assisted spine instrumentation (rLF) versus fluoroscopic freehand (fLF) instrumentation. This study looks to compare the early costs between matched lumbar rLF and fLF through (1) index surgery costs, including supplies used, length of surgery, and length of stay (LOS), and (2) revision surgeries secondary to complications or revisions within 3 months.

## Methods

### Patient Selection

After institutional review board approval was received (Institutional Review Board #2021-105), we retrospectively reviewed all patients undergoing robotic spinal fusion by one fellowship-trained senior spine surgeon at our institution and a matched cohort undergoing fluoroscopic freehand spine fusion by another fellowship-trained spine surgeon during the first-year adoption of the robot at the institution (2018 to 2021). All robotic procedures were conducted using the MAZOR X robotic system. Initial inclusion criteria included a robotic lumbar spinal fusion. Exclusion criteria included open spinal surgery and surgical history of posterior lumbar spine fusion. All included cases were minimally invasive/percutaneous surgeries. Patients meeting inclusion criteria were reviewed in depth for demographic data, body mass index (BMI), underlying diagnosis, spine approach, levels of fusion, LOS, length of surgery, 90-day readmissions, 90-day perioperative mortality, and 90-day revisions or returns to the OR.

Of the initial 73 rLF patients, some were unable to be confirmed as robotic cases (17) or had additional procedures conducted at the time of index fusion (6). Fifty patients met the final inclusion criteria. These robotic-assisted cases were matched to nonrobotic cases by the other spine surgeon for age (within 6 years), sex, approach to the spine (posterolateral interbody fusion and transforaminal lumbar interbody fusion only), and number of levels fused. Forty-two patients were matched. Cost data were available for 39 patients. Primary outcomes were day-of-surgery variable direct costs (VDCs). This included direct OR costs, supply costs, service costs, and LOS costs.

### Cost Data Acquisition

The term “costs” for this study refers to the actual VDC to the institution with each individual patient encounter. Costs were not the charges to the payor. The overall direct variable costs for each patient encounter included direct variable supply costs (including disposables) and other direct variable costs that included OR costs and costs related to time in the OR. Cost variables were acquired from the director of financial decision and support at our institution.

### Cost Definitions

Direct costs are thought to be a more accurate representation of true costs.^28^ For this study, total direct costs are the sum of the variable expenses directly related to patient care. VDCs represent incremental costs which would not have occurred if the surgery was not done. These costs vary with patient activity (ie, medications and medical tests). Included in these costs are labor wages for all personnel required for patient care of each surgical patient being treated, supplies (gowns, drapes IV equipment, etc, including robotic instruments), and drugs. By contrast, fixed direct costs (which were not evaluated in our study) represent incremental costs that would still have occurred even if the surgery was not done. This includes ongoing equipment costs (depreciation, maintenance contracts, and repairs), consulting fees, and administrative costs for both personnel (office manager, secretarial staff, etc) and office supplies/furnishings required to support this staff. Finally, the total direct costs were a summation of direct supply costs, direct costs of surgical time, and direct costs of services used. All variables were evaluated independent of the total cost and were compared between the approaches.

Capital investment for both fluoroscopic freehand and robotic setups was not included in our summaries. Indirect costs that were not directly related to individual patient care were not included in this study.

### Data Analysis

For analysis, we used Statistics Kingdom (statskingdom.com) and Past4 version 4.09 (University of Oslo). The Shapiro-Wilk test was used to assess the normality of the continuous variables. The categorical variables were analyzed using the Pearson chi square test for association, and the continuous variables were analyzed using the Mann-Whitney *U* test. For all analyses, a *P* value of <0.05 was considered statistically significant.

## Results

### Demographics and Clinical Outcomes

A total of 78 patients met criteria and were included in this study (39 rLF, 39 fLF—17 posterolateral interbody fusions, 22 transforaminal lumbar interbody fusions in each cohort). There were 54 one-level fusions, 18 two-level fusions, and six three-level fusions. Primary diagnoses in rLF included spondylolisthesis (32), degenerative disk disease (4), diskitis (1), disk herniation (1), and vertebral fracture (1). Primary diagnosis for fLF included spondylolisthesis (35), degenerative disk disease (1), spinal stenosis (1), and diskitis (1). No notable difference was observed in age, sex, BMI, history of lumbar spine surgery, OR time, or LOS (Table 1). rLF patients were more likely to have an American Society of Anesthesiologists rating of II (*P* = 0.04), and fLF patients were more likely to have an American Society of Anesthesiologists rating of III (*P* = 0.012). No significant difference was observed in 90-day readmissions between rLF and fLF, respectively (7.7% versus 2.6%, *P* = 0.305), or 90-day revision surgeries (7.7% versus 0%, *P* = 0.077). All readmissions in the robotic cohort underwent revision surgery; one patient had incomplete relief from an L4-L5 fusion (spondylolisthesis) and underwent an L3-L4 fusion; one patient had an interbody cage complication; and one patient had a surgical site infection. In the fluoroscopic cohort, one patient was readmitted for pain control because of sided radicular pain.

**Table 1:** Demographics and Clinical Outcomes

	rLF (39) Median ± SD	fLF (39) Median ± SD	*P* Value
Age	62.4 ± 10.2	62.4 ± 10.8	0.9722
Sex	22 female, 17 male	22 female, 17 male	1
BMI	30.6 ± 4.9	31.2 ± 5.1	0.7842
History of lumbar spine surgery	5 (12.8%)	3 (7.7%)	0.4554
ASA			
I	0	0	1
II	22	13	**0.04047**
III	15	26	**0.01262**
IV	1	0	0.3142
OR time (min)	172 ± 47.1	152.0 ± 39.1	0.1501
LOS	2.1 ± 1.7	2.1 ± 2.5	0.5194
90-d readmission related to surgery	3 (7.7%)	1 (2.6%)	0.6439
Revision surgeries within 90 d	3	0	0.07734

ASA = American Society of Anesthesiologists, BMI = body mass index, fLF = fluoroscopic freehand, LOS = length of stay, OR = operating room, rLF = robot-assisted spine fusion

### Total Encounter Costs

When comparing rLF with fLF, rLF had higher median total encounter costs ($23,122 ± 11,006 versus $18,328 ± 7,215, *P* = 0.009) and higher encounter VDCs ($15,867 ± 7,458 versus $13,580 ± 3,861, *P* = 0.001).

### Day of Surgery Costs

**Figure 1:**
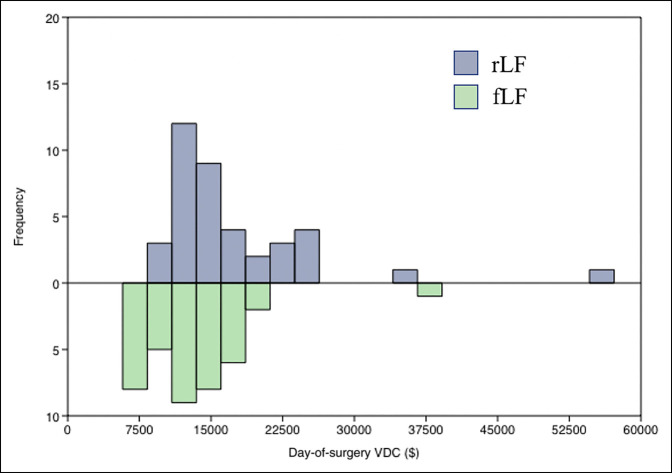
Graph showing day-of-surgery VDC distribution in rLF compared with fLF. fLF = fluoroscopic freehand, rLF = robot-assisted spine fusion, VDC = variable direct cost

**Figure 2:**
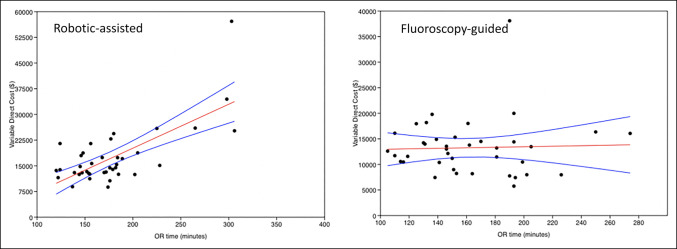
Graphs showing linear regression of OR time and day-of-surgery VDC for rLF (left) versus fLF (right). fLF = fluoroscopic freehand, OR = operating room, rLF = robot-assisted spine fusion, VDC = variable direct cost

rLF had higher day-of-surgery total VDCs ($14,444 ± 8,503 versus $13,012 ± 5,468, *P* = 0.005), including higher VDCs for anesthesia ($376.70 ± 354.87 versus $311.66 ± 157.00, *P* = 0.006), higher VDCs for supplies ($11367 ± 6914, *P* = 0.0183), and higher VDCs for OR time ($1,521 ± 1,246 versus $880 ± 454, *P* < 0.001) (Table 2 and Figure 1). No VDC differences were observed in imaging, laboratory test results, physical therapy/occupational therapy/speech services, room and board, postoperative recovery, and prescriptions. The three revision surgeries for the rLF cohort had an average day-of-surgery cost of $6571.80. Multivariate linear regression showed no relationship with age, BMI, or LOS with day-of-surgery VDCs for either rLF or fLF. OR time was similarly not related to cost for fLF. OR time did have a positive relationship with cost in rLF (r = 0.72, *P* < 0.001) (Figure 2).

**Table 2:** Total Encounter VDCs, Day-of-Surgery VDCs, and Revision VDCs

	rLF (39) Median ± SD	fLF (39) Median ± SD	*P* Value
Encounter total costs	$23,121.8 ± $11,006	$18,328 ± $7,215	**0.0091**
Encounter total VDCs	$15,867 ± $7457.8	$13,580 ± $3861	**0.001421**
Day-of-surgery costs			
VDC	$14,444 ± $8503	$13012 ± $5468	**0.005637**
Anesthesia	$376.70 ± $354.87	$311.66 ± $157	**0.006943**
Imaging (intraoperative)^a^	$83.11 ± $41.5	$68.49 ± $23.3	0.0602
Labs^a^	$39.63 ± $27.81	$47.64 ± $24.87	0.1837
Medical surgical supplies	$11,367 ± $6914	$9,931 ± $4,862	**0.01835**
OR time	$1,521 ± $1,246	$880 ± $454	**<0.001**
PT/OT/speech^a^	$24.64 ± $33.13	$30.74 ± $35.07	0.4324
Room and board^a,b^	$318.27 ± $267.08	$344.74 ± $499.32	0.77138
Recovery^b^	$226.53 ± $116.43	$224.98 ± $110.18	0.95192
Prescriptions	$342.66 ± $167.39	$374.74 ± $204.51	0.5689

fLF = fluoroscopic freehand, OR = operating room, rLF = robot-assisted spine fusion, VDC = variable direct cost

a PT/OT/speech—physical therapy, occupational therapy, speech therapy.

b Given as a mean and evaluated using the Welch *t*-test.

## Discussion

In our study, we found multiple factors that led to an overall increased cost in spine surgery with the use of rLF in comparison with fLF. Day-of-surgery cost and total encounter VDC were 11% and 16% more expensive, respectively, for the rLF group than the fLF group. Most of the difference between the two groups was found in the medical surgical supplies category, which had a 14% increase in cost for the robotic-assisted group.

### Clinical Outcomes

We found no difference in OR time or LOS between the cohorts. Readmissions and revision surgeries within 90 days were also similar but trended to have higher revision surgeries in the rLF group. The current literature indicates that the accuracy and precision with the use of robotics is highly effective, both in cadaver studies and patient cases.^29^ The complication rate was reduced in two studies reviewing robot-assisted MISs in comparison with fluoroscopy-guided surgeries,^3,30^ and there were few studies that indicated lower revision surgery and revision rates with the use of robotics.^3,31,32^ Kantelhardt et al^3^ reported a reduction in average length of hospital stay by 27%. However, a recent study by Passias et al^25^ showed a notable increase in postoperative complications in robotic surgeries, without change in revision rates. By contrast, a multicenter prospective study with 485 patients with 1-year follow-up between rLF and fLF found a 5.8× higher hazard ratio and 11.0× higher hazard ratio for fLF for complications and revisions, respectively.^33^ In our study, we did not find a correlation with less revisions and complications in the early adoption of robotic spine surgery. We aimed to look at short-term revisions and malpositioned screws that would necessitate early revision surgery.

### Costs

Our cost comparison showed several factors that led to an overall increased cost in spine surgery with the use of rLF in comparison with fLF. Most of the cost differences in the cohorts came from surgical supply utilization (14% increase with rLF) and OR time (72% increase with rLF). This also implies that there is a higher cost per minute in the OR with rLF. There was also a notable difference found in costs related to anesthesia, but this cost difference was minimal in comparison with the total VDC. Other fluctuations in price were also too minor to be impactful.

Very few studies discuss cost with most reviewing cost to the healthcare system but using measures other than VDC, in contrast to our study. Menger et al^26^ used a predictive model considering their caseload of 557 cases that resulted in an estimated savings of $608,000 over a 1-year period for one major academic center, with most of the savings coming from reduced infections, reduced revision surgeries, and decreased length of hospital stay. They used a “diagnosis-related group” as a basis for revenue calculations through reimbursement from Medicare or commercial insurances. Passias et al^25^ similarly looked at Medicare reimbursement and found that surgery costs were similar between open surgery and MIS, but robotic-assisted surgery resulted in the highest total cost. They also analyzed cost per quality-adjusted life year (QALY) as a main outcome measure, which showed robotic surgery to have the highest cost per QALY between groups, with MIS at the lowest cost per QALY. They also calculated the incremental cost-effectiveness ratio (a summary measure representing the economic value of an intervention), finding that MIS provided the highest effectiveness per cost ratio, followed by open surgery and then robotic surgery with the lowest ratio.

Garcia et al^27^ used point estimates and Monte Carlo methods to evaluate cost-effectiveness. Cost-effectiveness was found to be sensitive to OR/materials and admission costs. They also looked at the willingness-to-pay threshold per QALY and found that within the framework of $50,000/QALY, robotic-assisted MIS was cost-effective in 63% of simulations. The results from Kantelhardt et al^3^ showed that a reduction in the average length of hospital stay is possibly indicative of the cost-effectiveness of the use of robotics. Multiple studies also emphasized the reduction in surgery time based on the learning curve of a specific surgeon.^11,32^

Training spine surgeons to conduct robotic cases is another factor. Not all surgeons have access to this methodology as part of residency training. Future training may take a considerable amount of money, effort, and otherwise productive procedural or clinic time.^33,34^ Hu et al^4^ noted that the rate of success with robotic surgery improved after 30 procedures with a lower conversion rate to manual techniques.

Robotic-assisted surgery is slowly becoming more commonplace, and many studies do show some improved patient outcomes.^35^ Robotics in orthopaedics continues to grow, which may drive down the cost of larger cost variables, such as the overall price and maintenance of the robot. Although investing in the technology for robotic-assisted surgery may have a higher upfront cost, there is a cost-saving potential if it is found to consistently have fewer postoperative complications or revisions and decreased length of hospital stay. Data from our study imply that although the use of robotics is innovative and has possible positive patient-reported outcomes, the cost of implementation is currently higher to the healthcare system than its fluoroscopic-guided counterpart. If robotic-assisted spine surgery eventually leads to less revision surgeries or less infections as suggested in other literature, it would be important to evaluate postoperative costs over a longer time frame. Although our study indicates that the cost of robotic-assisted lumbar fusion procedures is currently higher than its fluoroscopic freehand counterpart, future research looking into the long-term postoperative costs across multiple healthcare systems would be beneficial.

There are other factors that play a role in the cost-benefit analysis of implementing robotic surgery in a specific institution. Hospital reputation with the use of a cutting-edge technology, patient perception of robotic surgery, and comfort of practicing surgeons, among other reasons, are various factors that may play a role in deciding whether introducing robotic surgery is worthwhile for a specific healthcare system.

### Robot Pricing and Maintenance

A portion of the cost that was not accounted for within the total costs of our study is the initial cost and maintenance of the robotics system. The cost of the MAZOR X robotic system is approximately $550,000 to $850,000.^24,35^ In addition, there may be yearly maintenance fees and an estimated disposable supply cost per case of $1,500.^24,35^

This study has a few limitations that are important to mention. The data are provided from one healthcare system within the United States. There may be a high degree of variability between costs between US institutions and especially between worldwide healthcare systems in labor costs, OR time, and medical supplies. Another limitation in this study is a relatively small sample size. After the inclusion and exclusion criteria were applied, the cases from 39 patients provided the only acceptable data for this study. We also did not have the cost of preoperative CT for rLF, nor labor, which has its own cost variation dependent on the institution. Another point of consideration is that while this study focused on the cost to the health system, the cost to the payor is also an important factor. The biggest strength of this study comes from details of our cost breakdown. This division allows for a better understanding of where these increased costs come from.

## Conclusion

Using VDC as our proxy for cost, the results from our study show that rLF is associated with a higher index surgery cost. The main factor driving increased cost is supply costs, with other variables too small in difference to have a notable financial effect. Future studies should focus on postoperative costs, including readmission and episode-of-care costs, and should consider the cost to the payor along with VDC. rLF will become more common, and other institutions may need to take a closer financial look at this more novel instrumentation before adoption.
